# Physical and functional interaction of the ciliopathy proteins Lrrc56 and Odad3 control deployment of axonemal dyneins in vertebrate multiciliated cells

**DOI:** 10.1242/dmm.052523

**Published:** 2025-12-19

**Authors:** Nayeli G. Reyes-Nava, Chanjae Lee, Ophelia Papoulas, Juyeon Hong, Edward M. Marcotte, John B. Wallingford

**Affiliations:** ^1^ Department of Molecular Biosciences, University of Texas at Austin, Austin, TX 78722, USA; ^2^ Department of Biological Sciences, Ulsan National Institute of Science and Technology, Ulsan 44919, South Korea

**Keywords:** Lrrc56, Cilia dynein, *Xenopus*, Ciliary dyskinesia

## Abstract

Primary ciliary dyskinesia (PCD) is a genetically heterogeneous motile ciliopathy characterized by chronic respiratory disease, laterality defects, hydrocephalus and infertility, caused by impaired function of motile cilia. *LRRC56* has recently emerged as a novel PCD candidate gene, but its role in vertebrate cilia remains poorly understood. Here, we used *Xenopus laevis* multiciliated cells, targeted knockdown and *in vivo* imaging to investigate *lrrc56* function, and combined these studies with *in vivo* affinity purification-mass spectrometry (AP-MS) to define its interactome. We show that loss of *lrrc56* causes specific depletion of outer dynein arms (ODAs) from the distal axoneme. *In vivo* AP-MS revealed that Lrrc56 binds the ODA docking complex components, including Odad3. Consistently, *lrrc56* knockdown also led to distal loss of Odad3. Moreover, we show that disease-associated variants in *LRRC56* and *ODAD3* disrupted their localization and interaction, pointing to a shared functional pathway. Our work demonstrates that *lrrc56* is a critical regulator of distal ODAs and ODA docking complex deployment and provides new mechanistic insight into PCD, advancing our broader understanding of motile cilia biology.

## Teaser

**Editor's choice:** This work defines a conserved role for Lrrc56 in outer dynein arm deployment in vertebrate multiciliated cells and identifies key protein interactions disrupted in ciliopathy-linked variants.

## INTRODUCTION

Motile ciliopathies are a group of inherited disorders caused by structural and/or functional defects in motile cilia, microtubule-based organelles that generate directional fluid flow across epithelial surfaces. These disorders affect multiple organ systems and are commonly associated with chronic respiratory infections, laterality defects, hydrocephalus and infertility (Wallmeier et al., 2020). Among them, primary ciliary dyskinesia (PCD) is the most extensively characterized at the genetic level, with more than 40 causative genes identified to date (Horani and Ferkol, 2021; Raidt et al., 2023). However, the clinical manifestations of PCD are heterogeneous and often overlap with other respiratory conditions, complicating diagnosis. Despite growing insights into the genetic underpinnings, many PCD-associated genes remain poorly characterized, and key molecular mechanisms underlying motile cilia dysfunction are still not fully understood.

**Fig. 1.**
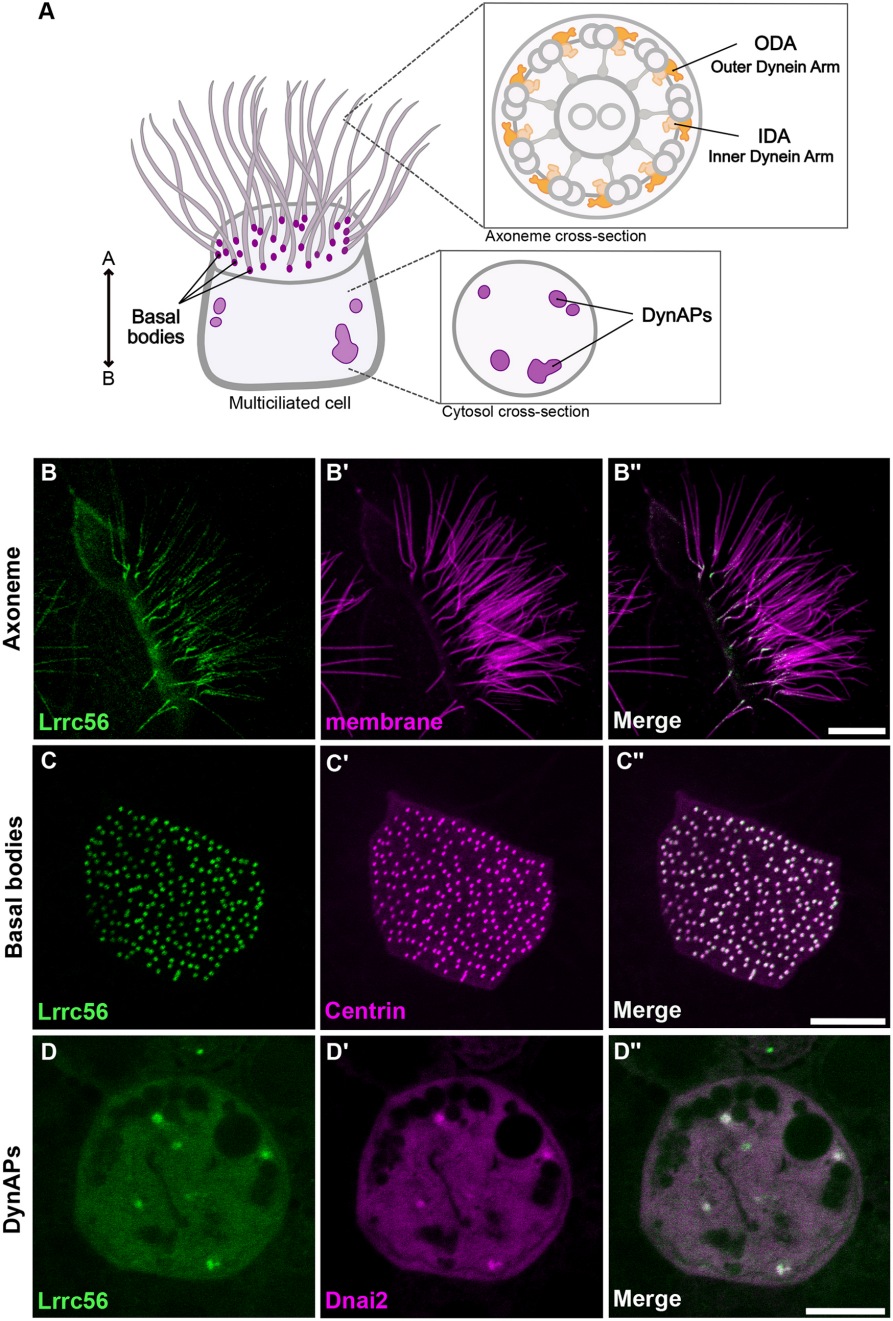
**Localization of GFP-Lrrc56 in *Xenopus* multiciliated cells.** (A) Schematic of a vertebrate multiciliated cell (MCC). The upper inset shows a cross-section of an axoneme with the relative positions of outer dynein arms (ODAs) and inner dynein arms (IDAs). The bottom inset illustrates a representative cross-section of the MCC cytoplasm, highlighting dynein axonemal particles (DynAPs). (B,C,D) Representative *in vivo* confocal images showing GFP-Lrrc56 (green) localization along the length of ciliary axonemes, basal bodies and at cytosolic foci (DynAPs) in *Xenopus* MCCs. (B′,C′,D′) Membrane labeling with CAAX-RFP (magenta) marks motile cilia, Centrin-RFP (magenta) marks basal bodies, and mcherry-Dnai2 (magenta) labels DynAPs. (B″,C″,D″) Representative merged confocal images from the previous channels showing GFP-Lrrc56 (green) and the various markers (magenta). Scale bars: 10 µm. For this experiment, the sample size was *n*=9 per group/condition. The experiment was independently repeated at least three times with consistent results.

Motile cilia beating is driven by axonemal dynein complexes such as outer dynein arms (ODAs), which line along the microtubule doublets to generate ciliary motion (King, 2016). ODA motors are preassembled in the cytoplasm (Fowkes and Mitchell, 1998) through the coordinated action of dynein axonemal assembly factors (DNAAFs) and chaperones (King, 2018; Qiu and Roy, 2022). ODA preassembly takes within specialized, membraneless organelles known as dynein axonemal particles (DynAPs), where DNAAFs, along with other assembly machinery, localize (Huizar et al., 2018) (Fig. 1A).

Following assembly, ODAs must be selectively and efficiently transported from DynAPs through the cytoplasm to the base of the cilium. This trafficking process is thought to involve specific transport adaptors and cytoplasmic motor proteins that mediate the handoff of ODAs to the intraflagellar transport machinery (Hibbard et al., 2022; Lechtreck, 2022). Once inside the cilium, ODAs are anchored to axonemal microtubules via the ODA docking complex (ODA-DC) (Gui et al., 2021). Critically, mutations in dynein subunits, DNAAFs or ODA-DC components disrupt this assembly and trafficking pipeline, resulting in motile ciliopathies (Bonnefoy et al., 2018; Leslie et al., 2022; Qiu and Roy, 2022). Although a few molecular players have been identified, the precise mechanisms by which ODAs are transported from DynAPs to the ciliary base remain poorly understood. Because proteins involved in dynein trafficking are frequently implicated in human disease, they present an entry point for understanding the mechanistic basis of these diseases.

One such protein, LRRC56, is a leucine-rich repeat protein, and its role in ODA transport and assembly was first proposed based on studies of its algal homolog ODA8 in *Chlamydomonas*. Variants in *LRRC56* have been associated with chronic respiratory disease and laterality defects in humans, consistent with the clinical presentation of PCD (Alasmari et al., 2022; Asseri et al., 2023; Bonnefoy et al., 2018). Surprisingly, despite these clinical phenotypes, transmission electron microscopy (TEM) of patient samples shows apparently intact ODAs (Bonnefoy et al., 2018). In *Trypanosoma*, loss of Lrrc56 disrupts ciliary motility and leads to selective loss of ODAs at the distal axoneme (Bonnefoy et al., 2018). More recently, murine studies show that Lrrc56 deficiency results in ciliary ultrastructural abnormalities and phenotypes consistent with PCD (Wu et al., 2025). Together, these findings highlight the clinical significance of *LRRC56* and the variability in ciliary ultrastructural phenotypes across species. Although work in unicellular models points to a role in ODA trafficking and distal targeting, how Lrrc56 functions in vertebrate cilia assembly and contributes to human disease remains an open and important question.

Here, we characterized Lrrc56 in vertebrate multiciliated cells (MCCs) and demonstrate its essential role in ODA deployment. In *Xenopus*, Lrrc56 localizes to DynAPs, basal bodies and axonemes. Importantly, ciliopathy-associated alleles in *LRRC56* disrupt its normal subcellular localization. Specifically, mutations in the leucine-rich repeat (LRR) domains and deletions of the intrinsically disordered regions (IDRs) result in distinct phenotypes, suggesting domain-specific functions for Lrrc56. Moreover, variants in *ODAD3* – homolog of an ODA-DC component identified here as a binding partner of Lrrc56 – not only alter its localization but also disrupt its functional interaction with Lrrc56. Together, these findings define a conserved role for Lrrc56 in vertebrate MCCs and provide mechanistic insight into the pathogenesis of *LRRC56*-related motile ciliopathies.

## RESULTS

### Lrrc56 localization pattern suggests a transport function

In ciliated unicellular organisms, like *Chlamydomonas* and *Trypanosoma*, Lrrc56 orthologs localize to both the flagella and cell body (Bonnefoy et al., 2018; Desai et al., 2015); however, their localization in vertebrate MCCs has yet to be described. We therefore turned to *Xenopus* embryo MCCs, which are highly amenable to live imaging and a reliable model of the biology of mammalian MCCs (Walentek and Quigley, 2017).

We found that GFP-Lrrc56 localized to motile cilia in *Xenopus* MCCs, displaying a punctate distribution along the axoneme with slight enrichment at the proximal end (Fig. 1B,B′). Additionally, Lrrc56 was strongly enriched at basal bodies, colocalizing with Centrin-BFP (Fig. 1C,C′), a pattern not previously reported in unicellular organisms.

ODAs are thought to be preassembled in DynAPs before being transported to cilia (Huizar et al., 2018), a process in which Lrrc56 has been implicated (Desai et al., 2015). We were interested, then, to observe that Lrrc56 was also enriched in foci in the cytoplasm (Fig. 1D). We confirmed that these foci reflect the ODA-specific region of DynAps (Lee et al., 2020) by co-labeling with the ODA subunit mCherry-Dnai2 (Fig. 1D′). Thus, Lrrc56 localizes to DynAPs, basal bodies and axonemes, consistent with its proposed role in trafficking of ODAs.

### *LRRC56* pathogenic variants associated with PCD disrupt its localization in MCCs

**Fig. 2.**
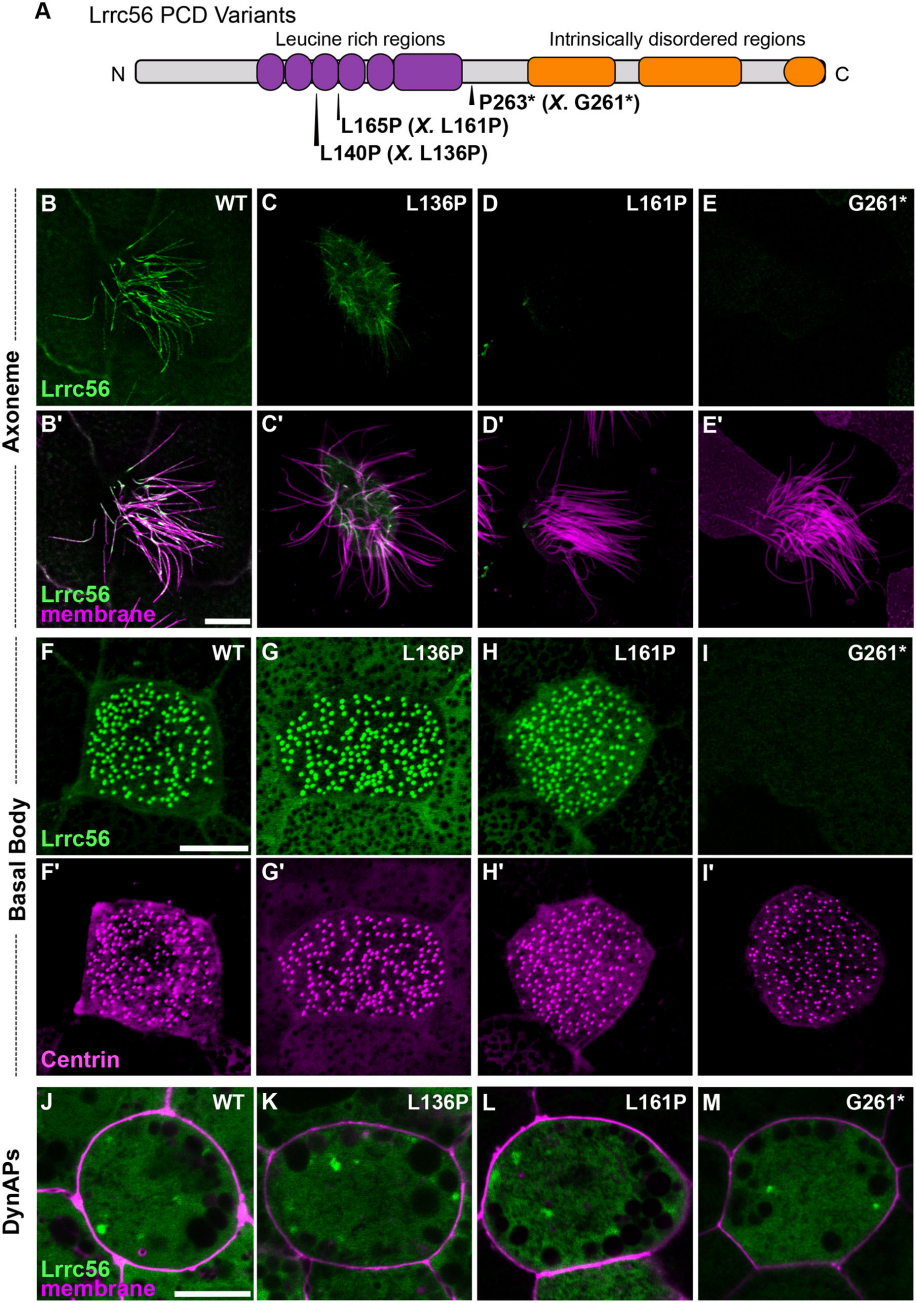
**Lrrc56 primary ciliary dyskinesia variants show a spectrum of localization defects.** (A) Schematic of LRRC56 (UniProt ID: Q8IYG6), showing the leucine-rich regions and long intrinsically disordered C-terminus. The location of three human primary ciliary dyskinesia (PCD) variants L140P, L165P and D266* is indicated. The corresponding *Xenopus* alleles are in parentheses and indicated by ‘*X*’. (B,C,D,E) *En face* images of single MCCs showing Lrrc56 (green) axoneme localization for wild-type (WT; B), L136P (human L140P; C), L161P (human L165P; D), and D261* (human P263*; E) variants. Lrrc56 axonemal localization is disrupted in all three variants. (B′,C′,D′,E′) Merged images showing Lrrc56 (green) with membrane labeling (magenta) reveal normal motile cilia structure. (F,G,H,I) Representative *en face* images of Lrrc56 basal body localization for WT (F), L136P (G), L161P (H) and G261* (I) variants. Variants located at leucine-rich domains show normal localization to basal bodies, but G261* variant fails to localize at basal bodies. (F′,G′,H′,I′) Centrin (magenta) labeling of basal bodies for the corresponding WT (F′), L136P (G′), L161P (H′) and G261* (I′) variants. (J-M) Merged channels showing Lrrc56 (green) and membrane (magenta) show cytosolic localization for WT (J), L136P (K), L161P (L) and G261* (M) variants. Scale bars: 10 µm. This experiment was independently replicated at least three times, with (*n*=3) randomly selected embryos from each batch used per replicate.

Pathogenic mutations in *LRRC56* have been implicated in PCD (Fig. 2A) (Alasmari et al., 2022; Asseri et al., 2023; Bonnefoy et al., 2018). Although one of these variants was linked to cilia motility in trypanosomes (Bonnefoy et al., 2018), the effect of these alleles in vertebrate MCCs is unknown. These disease variants affect residues conserved across species (Fig. S1), allowing precise modeling of the corresponding mutations in the *Xenopus* ortholog (Fig. 2A). To test whether ciliopathy alleles alter compartment-specific distribution, we generated the equivalent alleles in *Xenopus* Lrrc56 and expressed them in MCCs.

The L140P variant changes a well-conserved residue corresponding to *Xenopus* L136P within the LRR domain of Lrrc56 (Fig. 2A; Fig. S1). This variant showed reduced axonemal enrichment (Fig. 2C), while still localizing to basal bodies and DynAPs (Fig. 2G,K). Notably, this subcellular mislocalization contrasts with reports in *Trypanosoma*, in which the equivalent mutation did not alter Lrrc56 localization (Bonnefoy et al., 2018), highlighting potential species-specific differences in localization or function.

L165P – the most recurrent *LRRC56* variant found in PCD patients (Alasmari et al., 2022; Asseri et al., 2023) – is also well conserved (Fig. S1), and the homologous *Xenopus* L161P allele showed a complete loss of axonemal localization (Fig. 2D). Like L136P, this allele still accumulated at basal bodies and in DynAPs (Fig. 2H,L). Taken together, these two alleles suggest a requirement for the LRR domain in directing Lrrc56 to motile cilia.

Finally, we examined the P263* nonsense mutation (corresponding to *Xenopus* G261*), which truncates the C-terminal IDR of Lrrc56 (Fig. 2A; Fig. S1). Truncation of the IDR completely abolished Lrrc56 localization to axonemes and basal bodies (Fig. 2E,I). However, the G261* variant still localized to cytosolic foci (Fig. 2M; Fig. S2), although with reduced enrichment compared to wild type (WT) (Fig. S2). Western blot analysis confirmed that all variants, including G261*, are expressed at comparable levels to WT (Fig. S2), demonstrating that mislocalization is not due to protein instability or misfolding, but instead reflects a specific trafficking defect that prevents targeting to ciliary compartments.

Together, these findings reveal that pathogenic *LRRC56* variants disrupt Lrrc56 trafficking to motile cilia in distinct ways, suggesting that different domains of the protein contribute uniquely to its localization and function. While the LRR domains appear essential for axonemal targeting, the IDRs may play roles in cytosolic trafficking or basal body association. This work establishes a framework for dissecting the compartment-specific regulation of Lrrc56 – and how its mislocalization may contribute to PCD pathogenesis.

### Lrrc56 is essential for ODA deployment to motile cilia in *Xenopus*

Partial or complete loss of ODAs have been reported in unicellular organisms lacking Lrrc56, whereas *LRRC56*-mutant human respiratory cells show apparently normal axonemal ODA ultrastructure via TEM (Bonnefoy et al., 2018; Kamiya, 1988). Thus, to further explore the role of Lrrc56 in ODA deployment to axonemes in vertebrates, we performed loss-of-function analysis using knockdown of Lrrc56. An antisense morpholino (MO) oligonucleotide designed to block Lrrc56 splicing severely depleted the mRNA as indicated by reverse transcription PCR (RT-PCR) (Fig. S3).

**Fig. 3.**
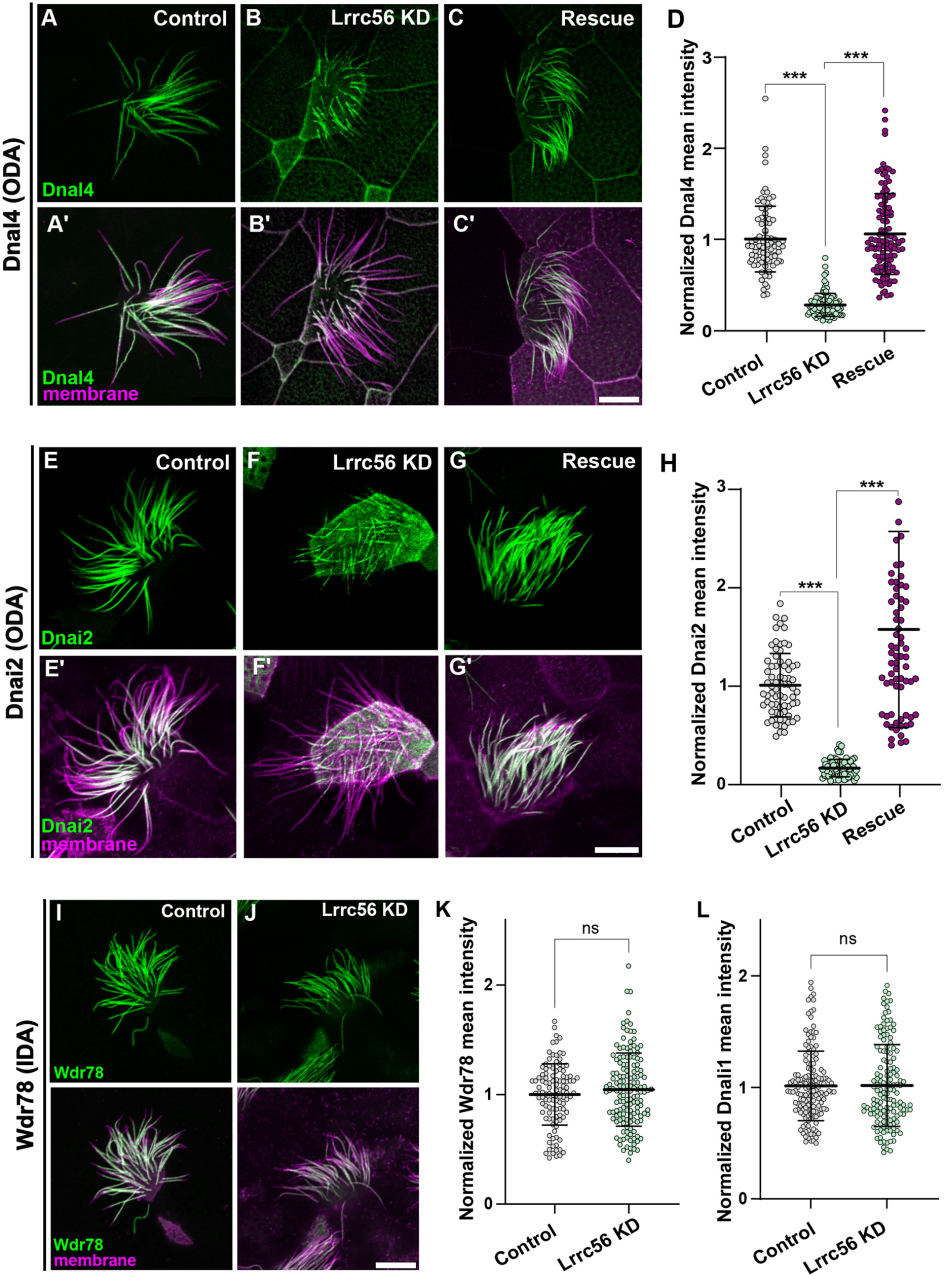
**Lrrc56 is essential for ODA deployment to axonemes.** (A) GFP-Dnal4 labeling of motile axonemes in control MCCs shows normal localization ODA subunits. (A′) Merged channels (membrane labeled with CAAX-RFP, magenta and Dnal4, green) reveal normal cilia morphology in control MCCs. (B) GFP-Dnal4 is absent from motile cilia in Lrrc56 knockdown (KD) MCCs. (B′) Membrane labeling (magenta) in merged channels shows normal motile cilia morphology despite Lrrc56 KD. (C) GFP-Dnal4 is restored to Lrrc56 KD motile cilia after ectopic expression of FLAG-Lrrc56. (C′) Merged channels show membrane (magenta) and Dnal4 (green) labeling in rescued MCCs. (D) Quantification of mean±s.d. of normalized GFP-Dnal4 fluorescence (see Materials and Methods) in axonemes of control (*n*=91), Lrrc56 KD (*n*=97) and rescue (*n*=11) MCCs. *N*>25 cells from nine embryos across three experiments for all conditions. (E,F,G) GFP-Dnai2 labeling of motile axonemes in control (E), Lrrc56 KD (F) and rescue (G) MCCs. GFP-Dnai2 shows normal localization in control MCCs but is lost from axonemes in Lrrc56 KD MCCs. Loss of GFP-Dnai2 is restored after ectopic expression of Lrrc56. (E′,F′,G′) Merged channels show membrane (magenta) and Dnai2 (green) labeling for cells in panels E, F and G, respectively. (H) Quantification of normalized GFP-Dnai2 mean intensity along axonemes in control (*n*=73), Lrrc56 KD (*n*=73) and rescue (*n*=74) MCCs. *N*>25 cells from nine embryos across three experiments for all conditions. (I) GFP-Wdr78 labeling of IDA subunits shows normal localization in motile axonemes. (J) GFP-Wdr78 remains localized to motile cilia in Lrrc56 KD MCCs. Merged channels (bottom row) show normal cilia morphology in both control and Lrrc56 KD MCCs, with membrane (magenta) and Wdr78 (green) labeling. (K,L) Graphs showing mean intensity of normalized IDA subunits Wdr78 (K) and Dnali4 (L) in control and Lrrc56 KD MCCs. Data are presented as mean±s.d, with error bars representing independent biological replicates. Statistical significance was determined using a non-parametric Mann–Whitney *U*-test for two-group comparisons and a one-way ANOVA for comparisons involving more than two groups. ***P<0.001; ns, *P*>0.05. Scale bars: 10 µm.

Lrrc56 knockdown disrupted the normal axonemal distribution of the ODA light chain subunit Dnal4 (GFP-Dnal4), which is typically enriched along the length of the axoneme (Fig. 3A,B). This defect was rescued by re-expression of Flag-tagged Lrrc56 (Fig. 3C), demonstrating the specificity of the knockdown. Quantification – normalized to a membrane-RFP marker – confirmed a significant reduction in Dnal4 levels in knockdown MCCs that was restored upon rescue (Fig. 3D). To confirm this result, we examined an additional ODA subunit, the intermediate ODA subunit Dnai2. We observed a similar phenotype, with Lrrc56 knockdown decreasing GFP-Dnai2 axonemal signal and rescue restoring it (Fig. 3E-H).

In human patients, inner dynein arms (IDAs) were unaffected after loss of LRRC56, and the same was true in unicellular organisms (Bonnefoy et al., 2018; Desai et al., 2015). Accordingly, we found that Lrrc56 knockdown did not disrupt the axonemal localization of the IDA subunits GFP-Wdr78 and GFP-Dnali1 (Fig. 3I-L). These findings support the conclusion that Lrrc56 is specifically required for deployment to axonemes of specific subunits of the ODA, but not IDA, in *Xenopus* MCCs.

### *In vivo* affinity purification-mass spectrometry reveals interaction between Lrrc56 and docking complex proteins

Although LRRC56 is linked to PCD, its molecular function remains unknown. We therefore sought to define the interaction landscape of Lrrc56 specifically in MCCs using *in vivo* affinity purification-mass spectrometry (AP-MS). We expressed GFP-tagged Lrrc56 under the control of an MCC-specific α-tubulin promoter (Deblandre et al., 1999) and dissected ∼750 ectodermal explants (‘animal caps’) from *Xenopus* embryos. Upon culture, these explants differentiated into mucociliary epithelium containing beating MCCs (Drysdale and Elinson, 1992; Walentek and Quigley, 2017). Protein was isolated and GFP-tagged Lrrc56 was immunoprecipitated using anti-GFP agarose beads. We performed the same method with GFP alone and subtracted the resulting background to calculate fold enrichment for Lrrc56-specific interactors (see Materials and Methods; Fig. S4, Table S1).

**Fig. 4.**
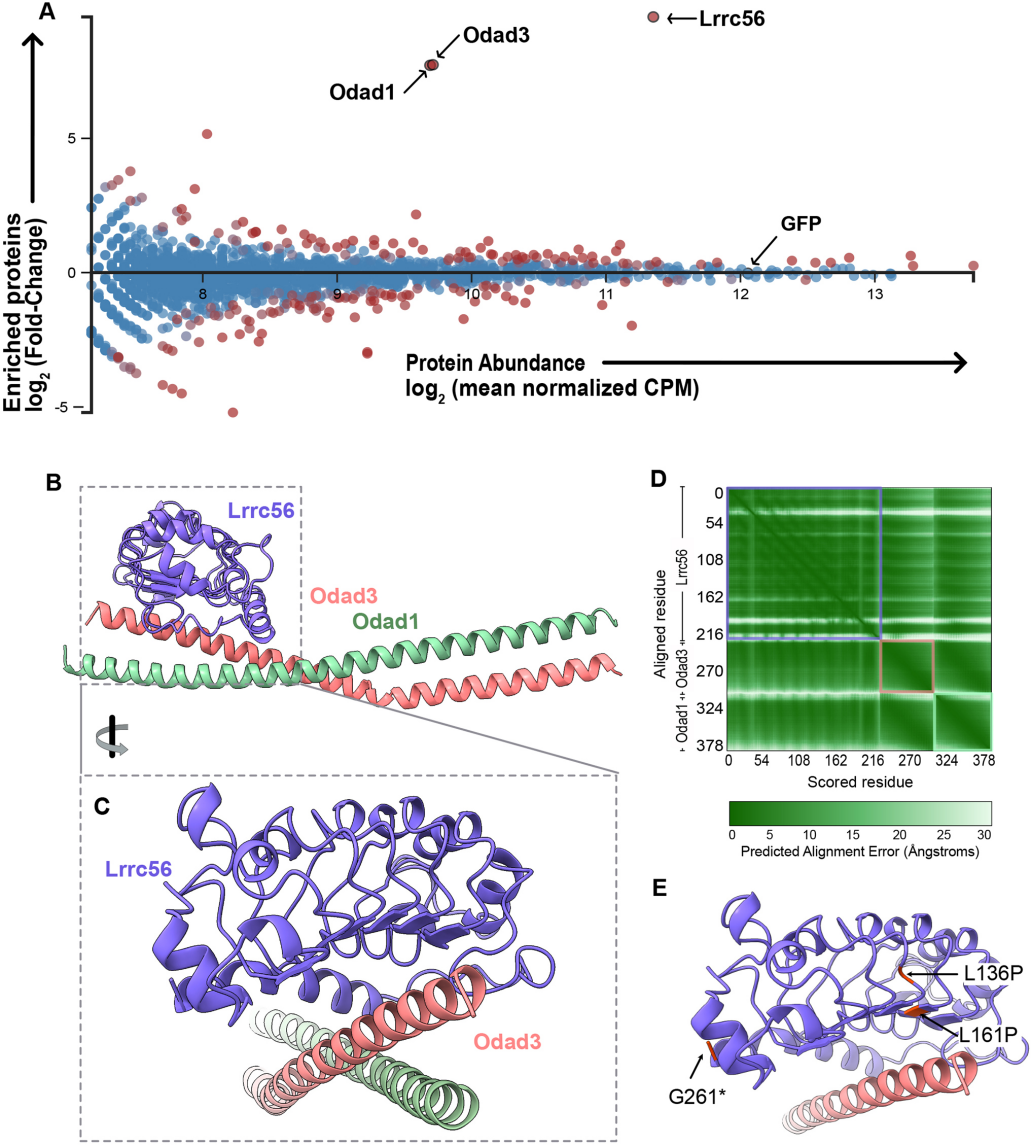
***In vivo* affinity purification-mass spectrometry reveals novel interactors of Lrrc56.** (A) MA plot of enriched proteins (log2 fold change) and average abundance between GFP-Lrrc56 (experiment) and GFP (control) immunoprecipitated proteins. Top hits are indicated with arrows. Lrrc56 (bait) is highly enriched and abundant in the experiment group. Odad1 and Odad3 are the top two enriched proteins in Lrrc56 IP group. GFP remains unchanged between control and experiment groups. MA plot was generated from two independent biological replicate experiments (see Materials and Methods). CPM, counts per million. (B) AlphaFold3-predicted structure of *Xenopus* Lrrc56 and its interactors, Odad3 and Odad1. Each monomer is color coded as indicated. The model includes residues 42-261 of Lrrc56, 162-239 of Odad3 and 137-220 of Odad1. (C) Enlarged view of the Lrrc56-Odad3 interface. (D) Predicted aligned error plot for the interaction between *Xenopus* Lrrc56, Odad3 and Odad1 in the model shown in B. (E) Close-up of the Lrrc56-Odad3 interface in the AlphaFold3 model, highlighting the positions of Lrrc56 ciliopathy-associated variants (L136P, L161P and G261*).

As expected, the bait protein Lrrc56 itself was the most strongly enriched hit, serving as a robust positive control (Fig. 4A). Moreover, our AP-MS analysis revealed strong enrichment of two key ODA-DC components: Odad1 (Ccdc114) and Odad3 (Ccdc151) (Fig. 4A). These coiled-coil proteins are central to the pentameric docking complex, recently resolved by cryo-electron microscopy of bovine respiratory cilia (Gui et al., 2021). The complex – composed of Odad1, Odad2 (Armc4), Odad3, Odad4 (Ttc25) and Odad5 (Calaxin) – forms a crucial bridge anchoring ODAs to the axonemal microtubule doublets (Gui et al., 2021; Yamaguchi et al., 2023). Importantly, mutations in *ODAD3* and *ODAD1* in humans are associated with motile ciliopathies, including PCD (Alsaadi et al., 2014; Hjeij et al., 2014; Knowles et al., 2013; Li et al., 2019). Moreover, loss of Lrrc56 is associated with disruption of docking complex machinery in *Trypanosomes* (Bonnefoy et al., 2024). Hence, co-enrichment of Odad1 and Odad3 with Lrrc56 strongly suggests a functional link between Lrrc56 and ODA docking machinery in vertebrate MCCs as well.

We therefore explored these interactions using protein structure modeling (see Materials and Methods; Fig. 4B). Initial AlphaFold3 modeling of full-length Lrrc56, Odad3 and Odad1 yielded a low-confidence multimer [interface predicted template modeling (ipTM)=0.41; predicted template modeling (pTM)=0.38], but the model had regions of high per-residue confidence scores [pLDDT (a per-atom confidence estimate on a 0-100 scale in which a higher value indicates higher confidence)>70] for the Lrrc56 LRR domain and associated coiled-coil regions of Odad3 and Odad1. These regions (residues 42-261 of Lrrc56, 162-239 of Odad3 and 137-220 of Odad1) were subsequently modeled in isolation, resulting in a higher-confidence structure (ipTM=0.74; pTM=0.69) (Fig. 4B,C). Consistent with these values, the predicted aligned error – a measure of AlphaFold3's confidence in the relative positioning of residue pairs – indicated a model with low global alignment error (Fig. 4D).

The ciliopathy-associated alleles L136P and L161P mapped to the core LRR domain of Lrrc56, positioned immediately adjacent to the predicted Odad3 interaction surface (Fig. 4E). These L>P changes, along with the altered backbone angles of P, are likely to disrupt local interactions, as well as potentially the folding of LRR globally. In contrast, the G261* truncation lies at the C-terminal end of the modeled region and removes the IDRs (not included in the structure), suggesting that these distal regions are critical for proper localization and potentially for interactions beyond the modeled core (Fig. 4E). Human LRRC56, ODAD3 and ODAD1 models were also generated and showed similar structures/folding, as well as localization of ciliopathy-associated alleles (Fig. S5).

### Lrrc56 is necessary for deployment of Odad3 to the distal end of the axoneme

**Fig. 5.**
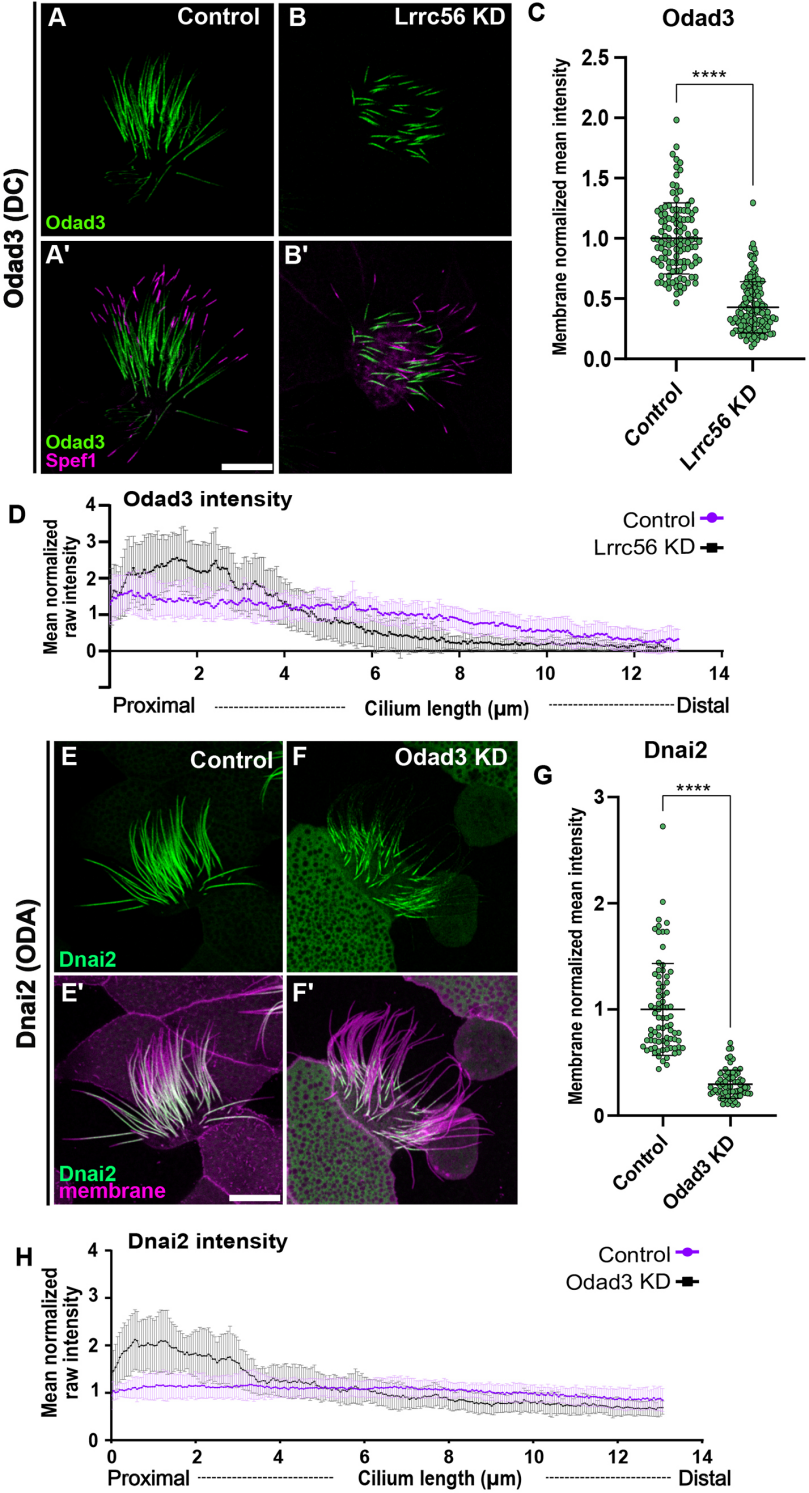
**Lrrc56 knockdown alters Odad3 localization at distal end of the motile axoneme.** (A) In control MCCs, the docking complex subunit Odad3 (green) localizes along the length of the motile cilium. (A′) Merged image showing Odad3 (green) and the distal tip marker Spef1 (magenta). Odad3 is enriched throughout the axoneme, excluding the Spef1-positive distal domain. (B) In Lrrc56 KD MCCs, Odad3 localization to axonemes is decreased. (B′) Merged image of Odad3 (green) and Spef1 (magenta) in Lrrc56 KD cilia shows reduced Odad3 intensity along the axoneme. (C) Quantification of normalized Odad3 mean intensity in control and Lrrc56 KD MCCs. Mean intensity was measured from the base of the cilium to the beginning of Spef1 domain in individual cilia; mean intensity was normalized to membrane marker; each dot represents a motile cilium. (D) Quantification of raw mean normalized Odad3 intensity along the axoneme in control and Lrrc56 KD MCCs. In Lrrc56 KD MCCs, Odad3 signal is enriched at the proximal end of the axoneme compared to controls (control, *N*=33; Odad3 KD, *N*=33). (E) The ODA subunit Dnai2 (green) localizes along the axoneme in control MCCs. (F) In Odad3 KD MCCs, Dnai2 intensity is reduced along the axoneme, particularly at the distal end. (E′,F′) Merged image of Dnai2 (green) and a membrane marker (magenta) in control (E′) and Odad3 KD (F′) MCCs. (G) Quantification of Dnai2 mean intensity in control and Lrrc56 KD MCCs, normalized to a membrane marker. (H) Quantification of Dnai2 intensity along the cilium in control and *odad3* KD MCCs. Plot shows representative axonemes (control, *N*=33; Odad3 KD, *N*=21). Statistical significance was determined using a non-parametric Mann–Whitney *U*-test. ****P<0.0001. Scale bars: 10 µm.

Because AlphaFold predicted a close interaction between Lrrc56 and Odad3, we asked whether disruption of Lrrc56 would affect Odad3 localization. In control MCCs, Odad3 localized along the length of the axoneme, excluding the most distal region, which is known to be enriched in Spef1 (Fig. 5A,A′). However, following Lrrc56 knockdown, the Odad3-GFP signal was significantly reduced along the axoneme compared to that in controls (Fig. 5B). To quantify this change, the mean intensity of Odad3-GFP along the axoneme was measured and normalized to a membrane marker. As shown in Fig. 5C, the mean Odad3-GFP intensity in Lrrc56-depleted MCCs was significantly decreased relative to that in controls.

Although the overall Odad3 signal was diminished, residual signal remained at the proximal region of the axoneme (Fig. 5B,B′). To further assess the distribution pattern, we quantified the raw Odad3-GFP intensity along the axoneme – excluding the Spef1-enriched distal domain – and normalized it to the mean Odad3-GFP intensity per cilium. In control cells, normalized Odad3-GFP intensity remained relatively constant along the first 8 µm of the axoneme (Fig. 5D). In contrast, in *lrrc56* morphants, Odad3 was strongly accumulated to above-normal levels within the proximal 4 µm (Fig. 5D), suggesting that impaired distal deployment of Odad3 is caused by loss of Lrrc56.

The specific distal loss of Odad3 prompted us to ask whether Lrrc56 contributes to ciliary tip organization, particularly the Spef1 domain. In control MCCs, the Spef1 domain typically measures ∼2-2.5 µm, whereas Lrrc56 morphants showed a modest increase to ∼2.8 µm (Fig. S6).

The pentameric ODA-DC is essential for the proper assembly of ODAs along the microtubules (Owa et al., 2014; Takada et al., 2002). Thus, we examined the impact of Odad3 knockdown on the distribution of the ODA subunit Dnai2, which is typically distributed along the entire motile axoneme in control MCCs (Fig. 5E,E′). As expected, Odad3 depletion resulted in substantial loss of ODA components in morphant MCCs (Fig. 5F,F′). Quantification of GFP-Dnai2 fluorescence mean intensity along the axoneme, normalized to a membrane marker, revealed a significant decrease in Dnai2 signal in *odad3* knockdown cells compared to that in controls (Fig. 5G). Notably, despite overall loss, Dnai2 accumulated at the proximal axoneme, reminiscent of Odad3 behavior in Lrrc56-deficient MCCs (Fig. 5B,B′). To capture this shift, we measured raw GFP-Dnai2 intensity along the axoneme and normalized it to the mean intensity in each condition. As shown in Fig. 5H, Dnai2 exhibited a proximal enrichment in Odad3-deficient cells.

### An Odad3 variant of unknown function disrupts interaction with Lrrc56

**Fig. 6.**
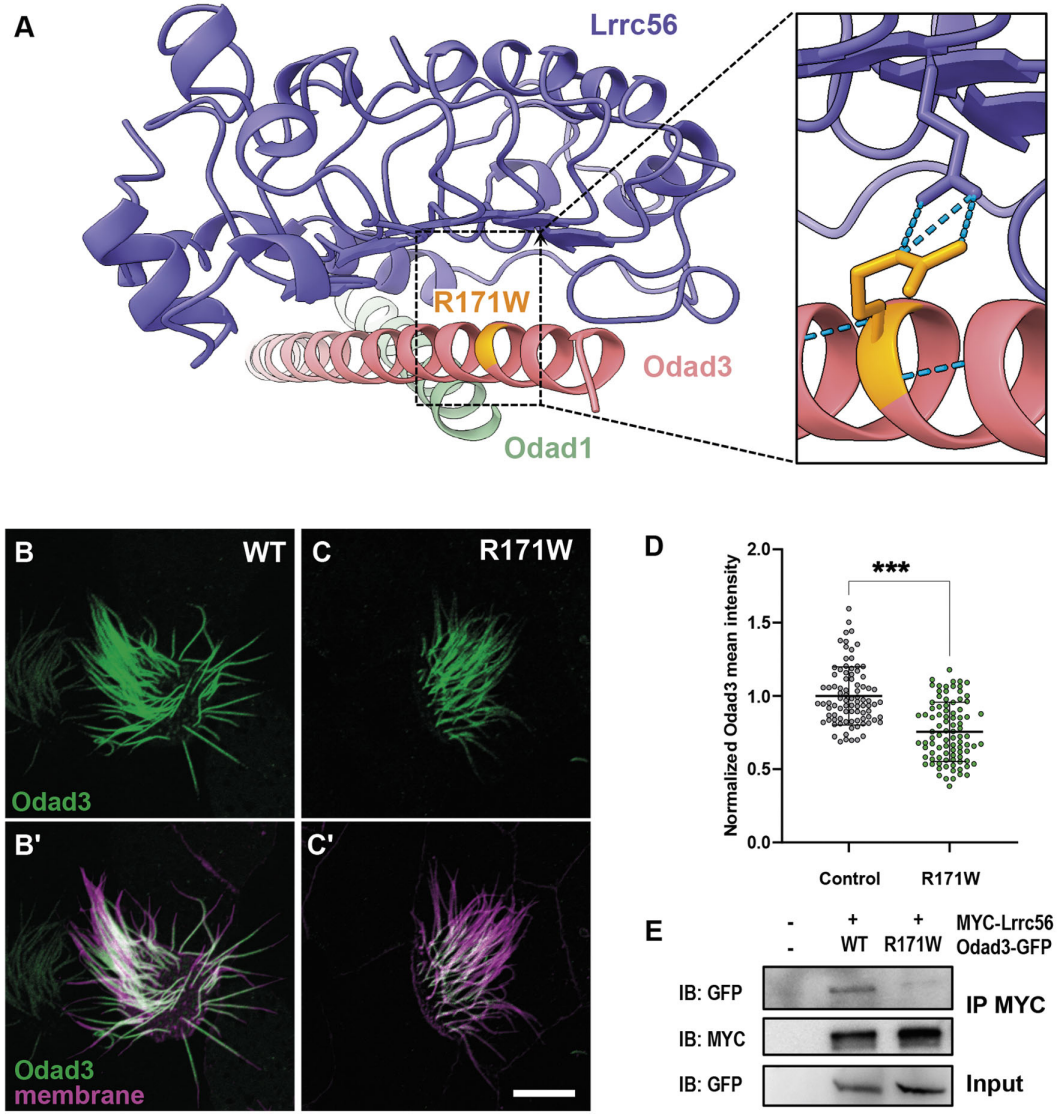
**An Odad3 variant of unknown function disrupts interaction with Lrrc56.** (A) AlphaFold3 model of *Xenopus* Lrrc56, Odad3 and Odad1, highlighting the location of the R171W (human E207W) variant of unknown function in Odad3. Inset shows a close-up of the Lrrc56-Odad3 interface, showing predicted interaction of R171 residue and Lrrc56 E96 residue. (B) WT localization of GFP-Odad3 in MCCs. Odad3 (green) localizes along the axoneme except for the most distal end of the cilium. (C) Expression of the R171W allele in MCCs disrupted its localization to axonemes compared to that in controls. (B′,C′) Merged image of Odad3 (green) and a membrane marker (magenta) in control (B′) and R171W allele (C′) MCCs. (D) Quantification of normalized mean Odad3 intensity along the motile cilium, showing decreased GFP signal R171W compared to that in controls (WT). Statistical significance was determined using a non-parametric Mann–Whitney *U*-test. ***P<0.0002. Scale bar: 10 µm. Three embryos (*N*=3) were analyzed; within each embryo, a minimum of three MCCs were imaged, and three to five individual cilia per cell were quantified. (E) Co-immunoprecipitation (IP) of Lrrc56 and Odad3. Representative western blot of IP of MYC-Lrrc56 and Odad3-GFP, and of input GFP, between WT and R171W allele. For total protein isolation, *n*=20 embryos per group were lysed in sample buffer.

A small number of validated PCD-causative variants in *CCDC151* (*ODAD3*) have been reported in the literature (Alsaadi et al., 2014; Asseri et al., 2023; Hjeij et al., 2014). However, many *ODAD3* variants of uncertain significance (VUS) are associated with ciliopathy in the human genetic database ClinVar. Notably, one such missense variant maps precisely to the Lrrc56-Odad3 interface (R207W) has been reported as a VUS in ClinVar (Fig. 6A).

This residue is well conserved in *Xenopus* (R171), so we examined its effect on localization in MCCs. Compared to controls (Fig. 6B,B′), the R171W variant of Odad3 displayed significantly reduced localization to the axoneme (Fig. 6C-D). Because this residue is predicted to be critical for interaction with Lrrc56, we assessed whether the R171W variant disrupts this interaction using co-immunoprecipitation assays. We co-immunoprecipitated Lrrc56 from animal cap explants and probed for WT or R171W Odad3. As expected, WT Odad3 co-immunoprecipitated with Lrrc56. However, the R171W variant showed reduced interaction with Lrrc56 compared to the WT protein (Fig. 6E). These findings demonstrate that a disease-associated variant of Odad3 disrupts its interaction with Lrrc56, potentially leading to defects in ciliary structure or function.

## DISCUSSION

*LRRC56* has recently emerged as a novel candidate gene implicated in PCD (Bonnefoy et al., 2018); but its functions have been explored largely in unicellular organisms. Here, we assessed the function of Lrrc56 specifically in vertebrates by leveraging the MCCs of *Xenopus* embryos and combining knockdown approaches with expression of human disease-associated variants and AP-MS to identify the interactome of Lrrc56. Our results reveal that Lrrc56 is required for the proper deployment of ODAs along MCC axonemes (Fig. 3B,F). Furthermore, we show that PCD-associated alleles impair protein localization in a variant-specific manner (Fig. 2B-M), suggesting distinct protein domain contributions to Lrrc56 function. Together, our study provides new mechanistic insight into *LRRC56*-associated motile ciliopathies and highlights *Xenopus* as a powerful platform to investigate the function of poorly characterized ciliary genes *in vivo.*

Using *in vivo* AP-MS, we identified Odad1 and Odad3 – core components of the ODA-DC – as candidate binding partners of Lrrc56. Whereas AP-MS does not distinguish between direct or indirect interactors, AlphaFold predicts a direct interaction between Lrrc56 and a heterodimer of Odad1 and Odad3. Notably, the position of the L>P disease alleles map to the predicted Lrrc56-Odad3 interface. This is consistent with direct binding. Moreover, a functional association between Lrrc56 and ODA-DC machinery has recently been reported in trypanosomes (Bonnefoy et al., 2024).

Similar to observations in *Trypanosoma brucei* (Bonnefoy et al., 2018, 2024), in which *Lrrc56* mutants exhibit loss of distal ODAs and ODA-DC during flagellar elongation, we observed that loss of Lrrc56 in *Xenopus* MCCs causes distal depletion of ODAs and ODA-DC components. Importantly, whereas in trypanosomes, Lrrc56 localization is restricted to the distal axoneme of growing cilia, our data in *Xenopus* MCCs show that Lrrc56 localization is not limited to the distal axoneme. Collectively, our results, and those of Bonnefoy et al. (2024), highlight conserved interactions between Lrrc56 and docking complex proteins, while also suggesting that aspects of Lrrc56 function are species dependent.

Studies in *Chlamydomonas* and *Trypanosoma* have uncovered proximodistal patterning of ODA-DC components, with distinct proximal and distal docking modules that contribute to ciliary beat regulation (Dutcher, 2020). In contrast, the mammalian ODA-DC proteins CCDC114 (ODAD1) and CCDC151 (ODAD3) are reported to distribute repeatedly along the axoneme (Gui et al., 2021). Our results on the differential accumulation of ODAs and ODA-DC components upon Lrrc56 loss suggest that vertebrate MCCs possess mechanisms for spatial regulation of docking complex activity along the ciliary proximodistal axis. These data raise the possibility that subtle proximodistal asymmetries exist but remain unresolved in vertebrate systems. The compartmentalized localization of Lrrc56 and its role in coordinating distal ODA and ODA-DC deployment may reflect such underlying specialization.

How ODA-DCs are trafficked from their synthesis site to the cilium remains elusive (Hibbard et al., 2022). Recent work in *Tetrahymena* has shown that the conserved protein Shulin binds cytoplasmic ODAs, stabilizing them into a compact, transport-ready conformation (Mali et al., 2021). By analogy, Lrrc56 may similarly function in the cytosol to stabilize or ‘package’ ODA-DCs for delivery to the ciliary base. Consistent with its localization to DynAPs, basal bodies and axonemes, which suggest a transport function, Lrrc56 may act to promote region-specific trafficking of ODA-DC machinery.

In summary, our work defines a conserved role for Lrrc56 in ODA deployment in vertebrate MCCs and identifies key protein interactions disrupted in disease-linked variants. By bridging insights from model organisms and human genetics, this study expands our understanding of motile ciliopathies and provides a framework for dissecting complex ciliary assembly pathways *in vivo*.

## MATERIALS AND METHODS

### *Xenopus* embryo manipulations

All *Xenopus* experiments were conducted in accordance with the animal protocol AUP-2024-00130 and the animal ethics guidelines of the University of Texas at Austin. Female adult *Xenopus laevis* were induced to ovulate by injection of human chorionic gonadotropin. *In vitro* fertilization was carried out by homogenizing a small fraction of a testis in 1/3× Marc's Modified Ringer's solution (MMR). Embryos were dejellied in 1/3× MMR with 3% (w/v) cysteine (at pH 7.8), and microinjected with mRNA or MOs in 2% ficoll (w/v) in 1/3× MMR. Injected embryos were washed with 1× MMR after at least 30 min and incubated in 1/3× MMR until the appropriate stages.

### Plasmid, mRNA and MO microinjections

*Xenopus* gene sequences were obtained from Xenbase. Open reading frames of genes were amplified from the *X. laevis* cDNA library by PCR and then inserted into a pCS10R vector containing a fluorescent tag. In addition to the vectors described previously (Huizar et al., 2018; Lee et al., 2020), the following constructs were cloned into pCS10R vector: GFP-Lrrc56, Flag-Lrrc56, MYC-Lrrc56, GFP-Odad1 and Odad3-GFP. To generate a *Xenopus* allele corresponding to the human patient allele, mutagenesis was performed on the GFP-Lrrc56 and GFP-Odad3 plasmids, using a Q5 Site-Directed Mutagenesis Kit (NEB, E0554S). All constructs were linearized, and the capped mRNAs were synthesized using a mMESSAGE mMACHINE SP6 transcription kit (Thermo Fisher Scientific, AM1340). Each mRNA was injected into two ventral blastomeres at the four-cell stage (∼30-80 pg per blastomere unless otherwise noted). For rescue experiments, 350 pg Flag-*lrrc56* mRNA per blastomere was injected. For AP-MS, GFP-*lrrc56* and GFP-empty plasmids (35 pg per blastomere) were injected into both ventral and dorsal blastomeres at the four-cell stage. MOs were designed to target the first or third exon-intron splicing junction of *lrrc56*, *odad3* and *odad1* in the allotetraploid genome of *X. laevis.* The MO sequences and the working concentrations included the following: *lrrc56* MO, 5′-ACTGAGTCTTAATGAAATCTTACCA-3′, 10-15 ng per injection; *odad3* MO, 5′-CCTTTAATCAACTGACTTACCCAGG-3′, 10 ng per injection.

### Live imaging and image analysis

Live imaging of multi-ciliated cells was performed in *Xenopus* embryos expressing tagged proteins at stages [Nieuwkoop and Faber staging (NF)] 24-26. Whole embryos were mounted between coverslips and immersed in 1/3× MMR prior to imaging. Imaging was conducted using a Zeiss LSM700 laser scanning confocal microscope equipped with a Plan-Apochromat 63×/1.4 NA oil immersion objective (Zeiss) or a Nikon Eclipse Ti confocal microscope with a 63×/1.4 NA oil immersion objective. For each experiment, three embryos (*N*=3) were analyzed. Within each embryo, a minimum of three MCCs were imaged, and three to five cilia per cell were quantified. Experiments were repeated across three biological replicates to ensure reproducibility. Quantitative image analysis was performed using Fiji. Graphs were generated, and statistical analyses, including error bars representing mean±s.d. and *P*-values, were conducted using Prism 10 software. Statistical significance was determined using a non-parametric Mann–Whitney *U*-test for two-group comparisons and a one-way ANOVA for comparisons involving more than two groups. Figures show all individual data points as well as mean±s.d. *P*≤0.05 was considered significant.

### Immunoprecipitation of *Xenopus* animal caps for mass spectrometry

To identify Lrrc56 interactors, circular plasmids of GFP only, or GFP-*lrrc56* driven by MCC-specific α-tubulin promoter were injected into four blastomeres of four-cell stage *Xenopus* embryos. Approximately 550 animal caps per sample were isolated at stage eight using forceps and were cultured in 1× Steinberg's solution [0.58 mM NaCl, 0.64 mM KCl, 0.33 mM Ca(NO_2_)_2_, 0.8 mM MgSO_4_, 5 mM Tris-HCl, 50 µg/ml gentamicin, pH 7.4-7.6] until sibling embryos reached stage 24. The cultured explants were collected, and immunoprecipitation was performed using a GFP-Trap Agarose Kit (ChromoTek, gtak-20). Immunoprecipitated proteins were eluted in 2× SDS-PAGE sample buffer (Bio-Rad, 1610747).

### AP-MS

Immunoprecipitated proteins in SDS-PAGE sample buffer were heated 5 min at 95°C before loading onto a 7.5% acrylamide mini-Protean TGX gel (Bio-Rad). After 7 min of electrophoresis at 100 V, the gel was stained with Imperial Protein stain (Thermo Fisher Scientific) according to the manufacturer's instructions. The protein band was excised, diced to 1 mm cubes and processed for in-gel trypsin digestion as in Goodman et al. (2018).

Digested peptides were desalted with 6 µg-capacity ZipTips (Thermo Fisher Scientific), dried, and resuspended in 20 µl of 5% acetonitrile, 0.1% acetic acid for mass-spectrometry. Peptides were separated using reverse-phase chromatography on a Dionex Ultimate 3000 RSLCnano UHPLC system (Thermo Fisher Scientific) with a C18trap to EASY-Spray PepMap RSLC C18 column (Thermo Fisher Scientific, ES902) configuration eluted with a 3% to 45% gradient over 60 min. Spectra were collected on a Thermo Fisher Scientific Orbitrap Fusion Lumos Tribrid mass spectrometer using a data-dependent top-speed high-energy-induced dissociation (HCD) acquisition method with full precursor ion scans (MS1) collected at 120,000 m/z resolution. Monoisotopic precursor selection and charge-state screening were enabled using advanced peak determination, with ions of charge+two selected for HCD with stepped collision energy of 30±3%. Dynamic exclusion was active for ions selected once with an exclusion period of 20 s. All MS2 scans were centroid and collected in rapid mode. Raw tandem mass spectrometry spectra were processed using Proteome Discoverer (v2.5) and the Percolator node to assign unique peptide spectral matches (PSMs) and protein assignments [false discovery rate (FDR) 0.01] to a *X. laevis* proteome derived from the 2023 UniProt *X. laevis* reference proteome of 35,860 protein sequences with homeologs and highly related entries collapsed into EggNOG vertebrate-level orthology groups (Huerta-Cepas et al., 2016). This database and the mass spectrometry data are available as MassIVE dataset MSV000098174.

In order to identify proteins significantly associated with each bait, we used the Degust statistical framework to calculate both a log2 fold change and an FDR for each protein enrichment based on the observed PSMs in the bait versus control pulldown. Settings used were ‘RUV (edgeR-quasi-likelihood), Normalization TMM, and Flavour RUVr’ and at least two counts in at least two samples.

### RT-PCR

To confirm the efficacy of Lrrc56 and Odad3 MOs, MOs were injected into all cells at four-cell stage of embryos. Total RNA was isolated with Trizol reagent (Invitrogen, 15596026) from *N*=4 embryos at stage 26, and cDNA was synthesized with an M-MLV reverse transcription kit (Invitrogen, 28025013). *lrrc56*, *odad3* and *odc1* were amplified by Taq-polymerase (Invitrogen, 10342020) with the following primers: Lrrc56.L 55F, 5′-gatttgggttggcaaggatta-3′; Lrrc56.L 508R, 5′-ctccagattgtttccctcaaga-3′; Odad3.L MO 63F, 5′-gcacaagaaactccagctcc-3′; and Odad3.L MO 367R, 5′-tcttcttctctgcctggtgg-3′.

### Immunoblotting

Embryos (*N*=20 stage NF 25) were lysed in lysis buffer (ChromoTek, gtak-20) supplemented with protease inhibitors. The lysates were centrifuged to remove cell debris, and the supernatants were subjected to SDS-PAGE followed by immunoblotting using standard protocols. The antibodies used were as follows: 1:200 anti-GFP antibody (Santa Cruz Biotechnology, sc-9996), 1:2000 HRP-conjugated goat anti-mouse IgG (H+L) secondary antibody (Thermo Fisher Scientific, 31430), 1:10,000 anti-β-actin monoclonal antibody (Proteintech, 6009-1) and anti-Myc antibody (9E10; Abcam, ab32).

## Supplementary Material

10.1242/dmm.052523_sup1 Supplementary information

Table S1. Table showing orthogroups and proteins with PSMs identified by APMS with Lrrc56.
