# Supplementary material for: Physical and functional interaction of the ciliopathy proteins Lrrc56 and Odad3 control deployment of axonemal dyneins in vertebrate multiciliated cells
Source: Dis Model Mech. 2025 Dec 19;18(12):dmm052523. doi: 10.1242/dmm.052523 (PMC12755065; doi:10.1242/dmm.052523)
Supplement: Supplementary information [file dmm-18-052523-s1.pdf]

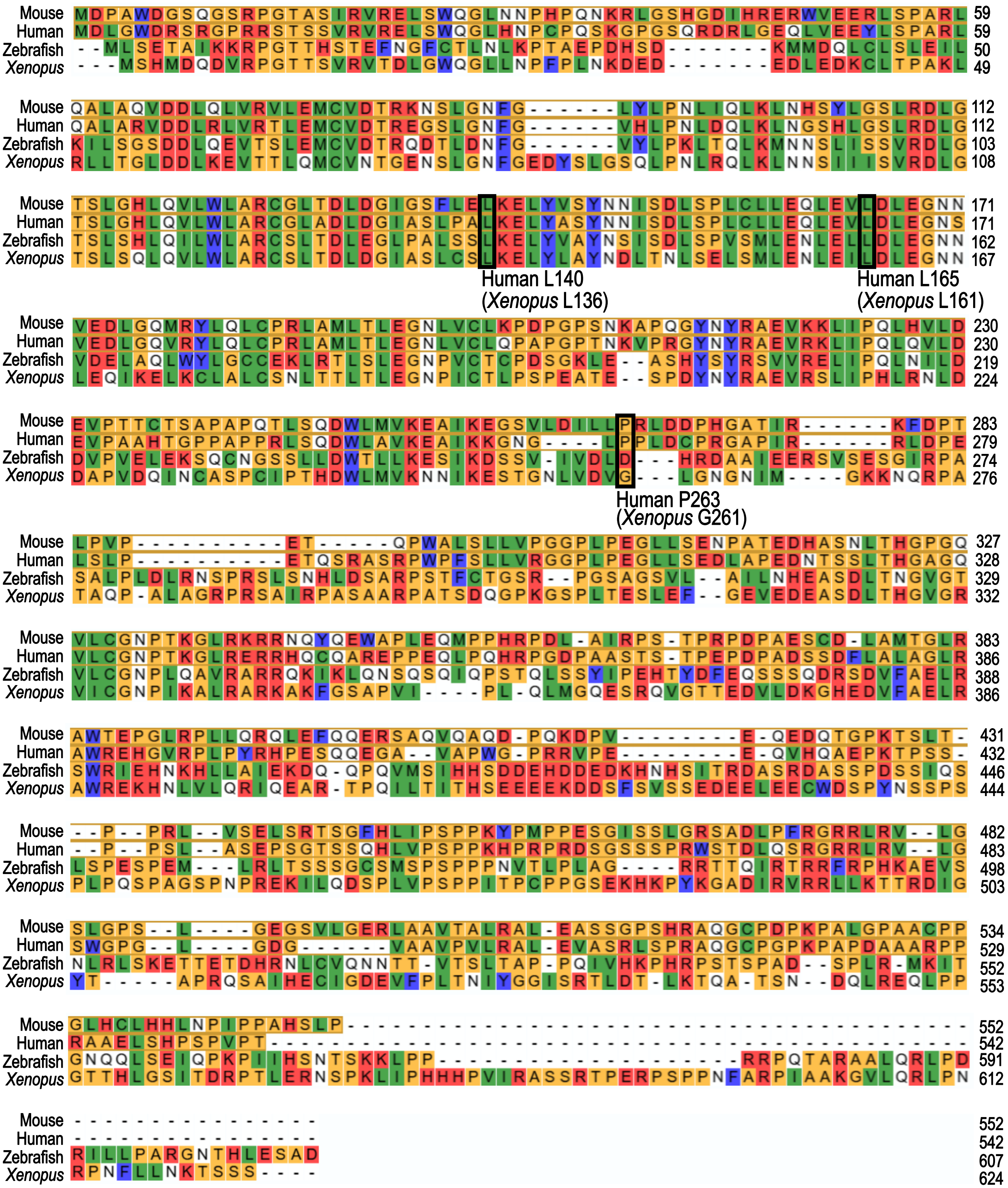
Mouse
Human
Zebrafish
Xenopus
Human L140
(*Xenopus* L136)
Human L165
(*Xenopus* L161)
Human P263
(*Xenopus* G261)
59 59 50 49
112 112 103 108
171 171 162 167
230 230 219 224
283 279 274 276
327 328 329 332
383 386 388 386
431 432 446 444
482 483 498 503
534 529 552 553
552 542 591 612
552 542 607 624

Fig. S1. Alignment of vertebrate Lrrc56 proteins. Multiple sequence alignment showing conserved Lrrc56 residues across mouse, human, zebrafish, and *Xenopus.* Rectangles indicate conserved ciliopathy loci characterized here. LRRC56 clustal analysis on mouse (Q8K375), human (Q8IYG6), zebrafish (A0A8M3APS4) and *Xenopus laevis* (A0A8J0V4N4). (UniProt ID). P263* allele results in a truncation of the LRRC56 IDR c-term region, Xenopus G261* is equivalent to this deletion.

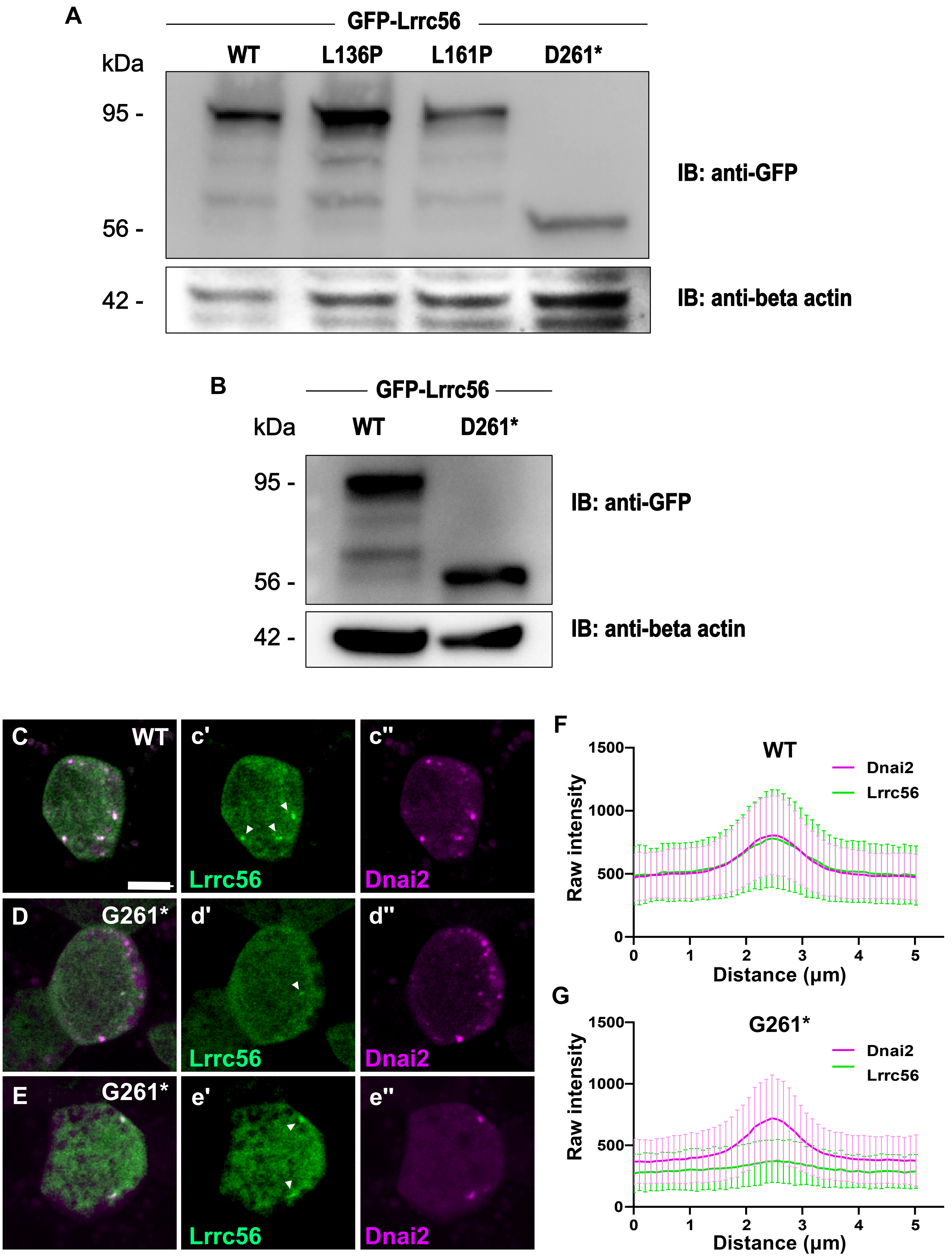

Disease Models & Mechanisms • Supplementary information

Fig. S2. Lrrc56 ciliopathy variants protein abundance.

Western blot showing protein levels for indicated disease alleles when expressed in *Xenopus* embryos. *Xenopus* variants (human): L136P (L140P), L161P (L165P), G261* (P263*). Western blot of total protein from N=20 embryos NF 25, injected with 80pg of GFP-Lrrc56 WT, L136P, L161P and 160pg of D261*. Anti GFP 1:200 and Anti B-actin housekeeping control (1:10,000).

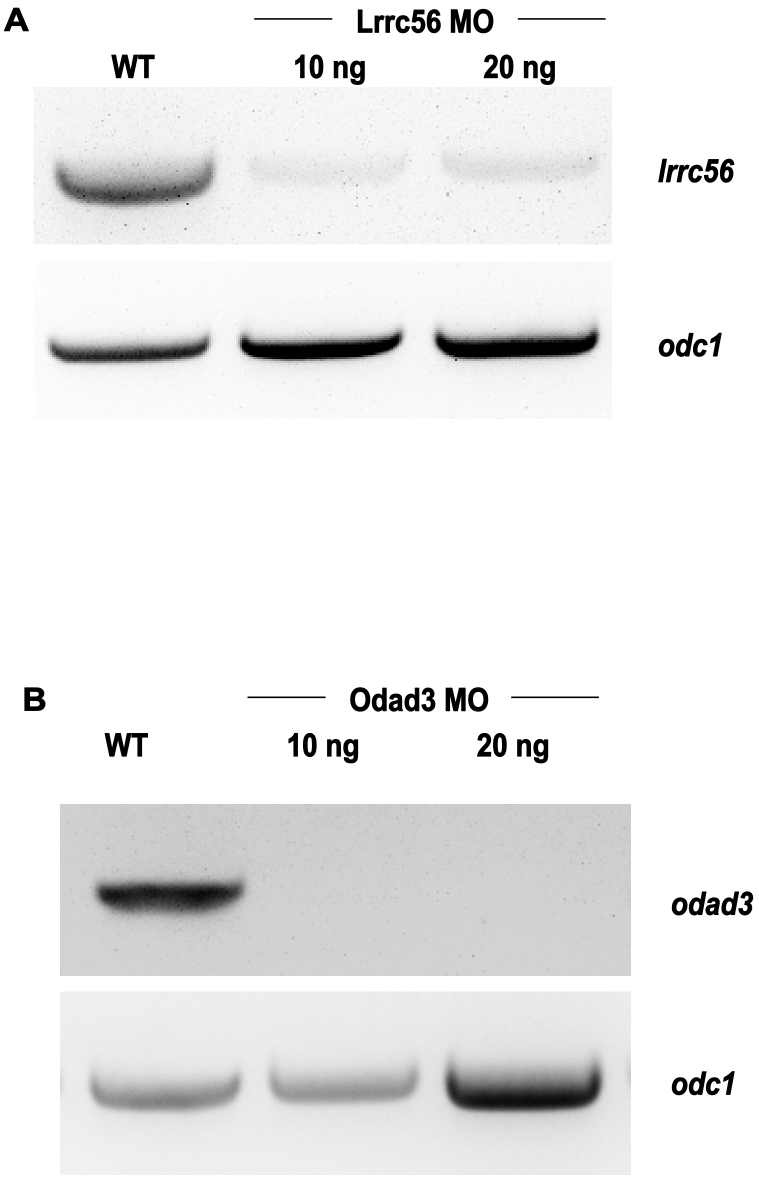

Fig. S3. Validation of Lrrc56 and Odad3 splice-blocking morpholino efficiency by RT-PCR. (A) Gel image of RT-PCR of *lrrc56* and *odc1* mRNA levels in wildtype control (WT), Lrrc56 MO 10ng and 20ng injected embryos. (B) Gel image of RT-PCR of *odad3* and *odc1* mRNA levels in wildtype control (WT), Lrrc56 MO 10ng and 20ng injected embryos.

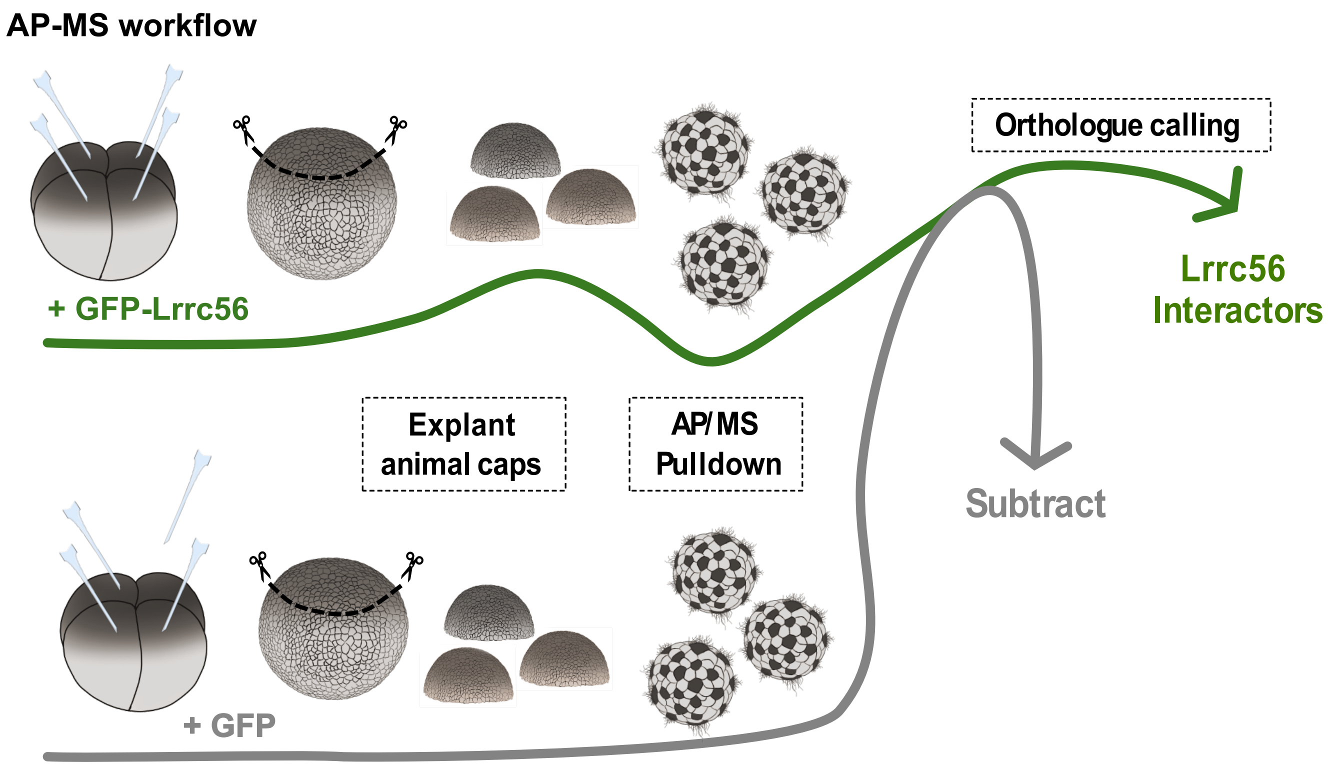

Fig. S4. Schematic of AP-MS workflow for identification of *in vivo* Lrrc56 interactors. A plasmid encoding GFP-tagged Lrrc56 under the control of the MCC-specific α-tubulin promoter was injected into Xenopus embryos at the 2–4 cell stage (stage 3). Animal cap explants were dissected at stage 8 and cultured until the early stage of ciliogenesis (stage 23). Explants were then harvested and subjected to GFP-based immunoprecipitation followed by affinity purification mass spectrometry (AP-MS). A parallel experiment using unfused GFP was performed to account for non-specific interactions, and these were subtracted from the experimental dataset to identify specific Lrrc56 interactors.

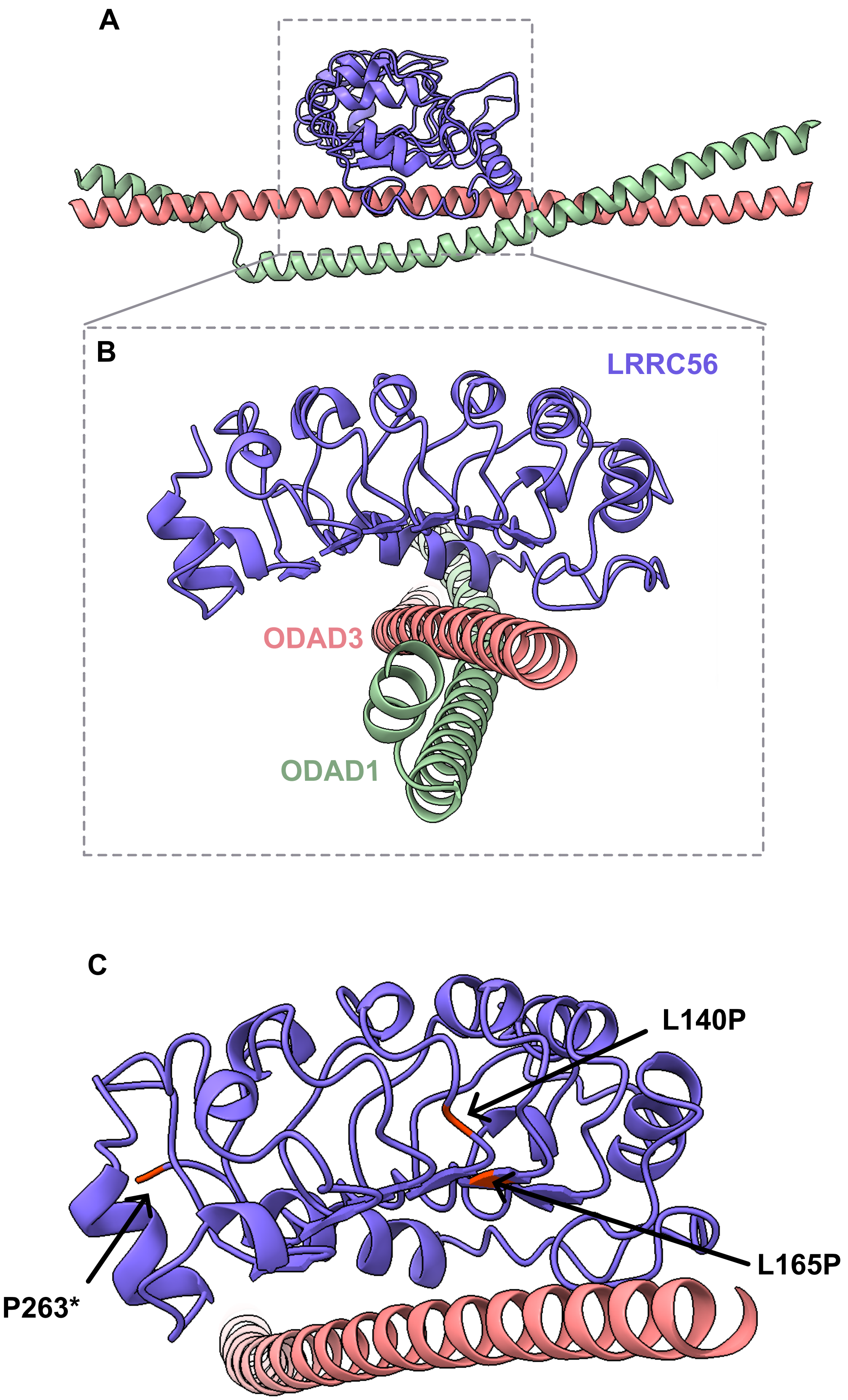

Disease Models & Mechanisms • Supplementary information

Fig. S5. Human AF3 model of LRRC56, ODAD3 and ODAD1.

(A) AlphaFold3-predicted structure of human LRRC56 and its interactors, ODAD3 and ODAD1. Each monomer is color-coded as indicated. The model includes residues 53-263 of LRRC56, 159-315 of ODAD3, and 80-224 of ODAD1.

(B) Enlarged view of the LRRC56–ODAD3 interface

(C) Close-up of the LRRC56–ODAD3 interface in the AlphaFold3 model, highlighting the positions of Lrrc56 ciliopathy-associated variants (L140P, L165P, and P263*).

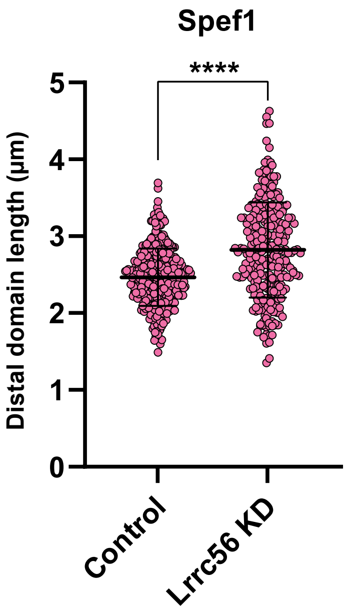

Fig. S6. Spef1 domain quantification in Lrrc56 KD.

Quantification of Spef1 domains in control and Lrrc56 knockdown (KD) multiciliated cells. The length of the Spef1-positive distal domain per cilium was measured from confocal images. Each dot represents an individual Spef1-domain measurement. Data are presented as mean ± SEM; Control: 352 cilia from 30 cells across 5 embryos, Lrrc56 KD: 277 cilia from 39 cells across 8 embryos, from 2 independent experiments per condition. Statistical analysis was performed using the Mann–Whitney test.

Table S1. Table showing orthogroups and proteins with PSMs identified by APMS with Lrrc56.

Available for download at
https://journals.biologists.com/dmm/article-lookup/doi/10.1242/dmm.052523#supplementary-data

Disease Models & Mechanisms • Supplementary information
